# Perceived ageism and psychosocial outcomes during the COVID-19 pandemic

**DOI:** 10.1186/s13690-024-01297-2

**Published:** 2024-05-10

**Authors:** André Hajek, Hans-Helmut König

**Affiliations:** Department of Health Economics and Health Services Research, University Medical Center Hamburg-Eppendorf, Hamburg Center for Health Economics, Martinistr. 52, 20246 Hamburg, Germany

**Keywords:** Ageism, Loneliness, Social isolation, Social exclusion, Depressive symptoms, Life satisfaction

## Abstract

**Background:**

In light of the existing knowledge gap in this research area (particularly based on representative samples and research conducted during the pandemic), the objective of this study was to explore the association between perceived ageism and psychosocial outcomes (i.e., in terms of life satisfaction, loneliness, social isolation, aging satisfaction and depressive symptoms) among middle-aged and older adults during the COVID-19 pandemic based on nationally representative data.

**Methods:**

We used data from the nationally representative German Ageing Survey, which covers community-dwelling middle-aged and older individuals aged 40 years or over. Specifically, wave 7 of the German Ageing Survey (conducted from November 2020 to March 2021) was analyzed, consisting of a sample of 4,167 individuals with an average age of 68.7 years (SD: 10.1 years; ranging from 46 to 98 years). Established instruments were employed to measure psychosocial outcomes.

**Results:**

Regressions showed that the presence of perceived ageism was significantly associated with unfavorable psychometric outcomes (i.e., higher loneliness: β = 0.29, *p* < 0.001; higher perceived social isolation: β = 0.32, *p* < 0.001; more depressive symptoms: β = 2.68, *p* < 0.001; lower life satisfaction: β=-0.28, *p* < 0.001; higher negative affect: β = 0.21, *p* < 0.001; lower aging satisfaction: β=-0.19, *p* < 0.001), except for positive affect (β=-0.06, *p* = 0.10). Stratified by age group (i.e., individuals 40 to 64 years; individuals aged 65 years and over, see Tables 3 and 4), comparable results were obtained in terms of significance.

**Conclusions:**

Perceived ageism was associated with unfavorable psychosocial outcomes. This knowledge can help reduce vulnerability to negative psychosocial factors in people in the middle and later years of life.

**Supplementary Information:**

The online version contains supplementary material available at 10.1186/s13690-024-01297-2.

**Table:** 

Text box 1. Contributions to the literature
• There is a small number of quantitative studies (based on generalizable samples) examining the association between perceived ageism and psychosocial factors, particularly during the pandemic.
• We aimed to address this gap in knowledge and found that perceived ageism was associated with unfavorable psychosocial outcomes.
• This understanding can help to reduce vulnerability to negative psychosocial outcomes in individuals in the middle and later stages of life.

## Background

Perceived ageism refers to subjective experiences of prejudices and discrimination against individuals based on their age. It is a frequent challenge [1]. Former research particularly stressed the importance of perceived ageism on physical health (see these recent systematic reviews as an overview: [2, 3]). However, there is restricted knowledge regarding the association between perceived ageism and psychosocial outcomes (e.g., including factors such as social isolation, depressive symptoms, or life satisfaction).

More precisely, some quantitative studies have examined the association between perceived ageism and psychosocial factors. A recent systematic review summarized the existing studies [4] and stressed “that the research on the relationship between ageism and the psychological well-being of older adults is at an early stage with ample room for development” (p. 16). They also emphasized the small number of quantitative studies in this area and highlighted the need for more quantitative studies based on generalizable samples [4].

For example, Avidor et al. [5] found an association between perceived ageism and subsequent lower subjective well-being (covering life satisfaction, positive and negative affect) among middle-aged individuals. An association between perceived ageism and lower life satisfaction has also been observed in an online survey with limited generalizability [6]. Another study based on a convenience sample of 30 women also identified an association between hostile ageism and lower life satisfaction among women aged 60 years and over in three districts of Odisha (India) [7]. More broadly (and apart from perceived ageism), former research demonstrated an association between perceived discrimination and lower well-being among immigrants in Italy [8]. Another study also revealed an association between perceived discrimination and mental distress in Vietnamese Americans [9]. A former meta-analysis also identified an association between perceived discrimination and psychological well-being [10].

Given the existing knowledge gap in this research area (particularly based on *representative samples* and research conducted *during the pandemic*), our aim was to explore the association between perceived ageism and psychosocial outcomes (i.e., in terms of life satisfaction, loneliness, social isolation, aging satisfaction and depressive symptoms) among middle-aged and older adults in the particular context of the COVID-19 pandemic based on nationally representative data.

We cautiously assume that middle-aged adults may be more sensitive to (perceived) ageism as they are confronted with societal expectations to maintain youthfulness and it may be a new stressor for them, which both may lead to increased stress, lower general self-esteem, and dissatisfaction. Moreover, middle-aged adults are frequently in paid employment. Thus, perceived ageism may have an impact on their career and income. This could markedly contribute to their psychosocial well-being. Older adults, in contrast, who may have adapted to their ageing identity and may be less reliant on social affirmation through work or the social sphere, may show greater resilience to ageist attitudes. This could lead to less pronounced psychosocial effects.

In our view, it is important to investigate the association between perceived ageism and psychosocial factors. Such knowledge may assist in clarifying the factors associated with psychosocial outcomes and can help to address individuals at risk for adverse psychosocial factors. Moreover, such knowledge may help to raise awareness of the detrimental factors associated with perceived ageism and may thus promote changes within society, i.e., a more inclusive and age-friendly society.

With regard to the theoretical background of this study, several theories were proposed to study the association between ageism and psychosocial factors (as an overview: [4]): for instance, stereotype embodiment theory [11], or the stress process model [12]. In accordance with the stress process model [12], repeated encounters with ageism can act as stressors, and the exposure to such distressing experiences may increase, among other things, depressive symptoms [12]. The stereotype embodiment theory [13] emphasised that the internalisation of age stereotypes occurs through people because they are exposed to them throughout their lives. They begin to adopt and display the characteristics and behaviours associated with the stereotype - self-stereotype. According to this theory, such internalised stereotypes can have actual consequences (both conscious and unconscious) for one’s own well-being and health [13].

With regard to the association between perceived ageism and psychosocial factors *during the pandemic*: A former study showed that ageism markedly increased during the pandemic [1]. Numerous specific cases of ageism were identified in newspapers worldwide, providing detailed examples of discriminatory attitudes towards older individuals [14]. For instance, it has been proposed that individuals in old age should self-sacrifice for the benefit of the younger generations and the economy (see also: [15]). The other extreme focused on the vulnerability of older people - and their absolute protection at all costs [16]. Thus, against the backdrop of the increase in ageism, it appears very important to examine the association between perceived ageism and psychosocial outcomes.

## Methods

### Sample

Data for this study were obtained from wave 7 of the German Ageing Survey (DEAS, “Deutscher Alterssurvey”), which included individuals aged 40 years and above residing in the community.

The German Aging Survey received funding from the Federal Ministry for Family Affairs, Senior Citizens, Women, and Youth (BMFSFJ). Fieldwork for the survey was carried out by a reputable company known as infas, conducted from November 2020 to March 2021.

It may be worth describing the conditions of the COVID-19 pandemic in Germany prior and particularly during the time of data collection: Nationwide measures to combat the transmission of COVID-19 were initiated in mid-March 2020, including the temporary closure of schools. Some of these restrictions were eased in mid-April 2020, allowing schools to reopen in May 2020. However, when infection rates began to rise again in autumn 2020, new restrictions were imposed. Then, in May 2021, certain restrictions were lifted to allow a gradual return to normality.

The survey employed a Computer-Assisted Personal Interviewing (CAPI) method conducted over the phone. In addition, participants were provided with a printed drop-off questionnaire for self-completion, which included more sensitive questions (e.g., psychosocial factors). The average duration of an interview in wave 7 was approximately 75 min. The primary aim of the DEAS study is to examine a wide range of significant aspects related to later life, including but not limited to workforce engagement, well-being, health, and perceived ageism.

The DEAS study utilizes a cohort-sequential design. Due to the limitations imposed by the COVID-19 pandemic, it was not possible to conduct face-to-face interviews for a survey. Moreover, it was not possible to introduce a new baseline sample due to the pandemic. Thus, the sample for wave 7 consisted of individuals who remained accessible and willing to participate in the panel from the baseline samples spanning from 1996 to 2014. The overall gross sample comprised nearly 8,400 individuals, of which 5,402 individuals aged between 46 and 100 had valid interviews available, resulting in a response rate of approximately 65%. Furthermore, 4,419 participants (82% of the total) completed the written drop-off questionnaire. The analytical sample consisted of *n* = 4,167 individuals (with loneliness as outcome measure), accounting for some missing values. The primary reason for non-participation was the general refusal to participate and the linked withdrawal of willingness to be part of the panel. However, factors such as community size (i.e., grouping communities based on their population size such as under 2,000 inhabitants, 2,000 - and 5,000 inhabitants and so on), income category and family status were mainly not related to the participation likelihood [17]. Additional details regarding the DEAS survey was provided by Klaus et al. [18].

Prior to the interview, each participant provided written informed consent. The DEAS study adheres to the principles outlined in the Helsinki Declaration. As the study did not meet the necessary criteria for ethical approval, such as not posing risks to respondents or lacking information about the study’s objectives, obtaining ethical approval was not deemed necessary for the DEAS study.

### Dependent variables

Bude and Lantermann [19] developed a scale to evaluate social isolation, which consisted of four items rated on a scale from 1 (strongly agree) to 4 (strongly disagree). All items were recoded, and the average rating across all items was calculated, with higher values indicating greater perceived social isolation. In our study, Cronbach’s alpha was found to be 0.87 (McDonald’s omega: 0.88).

For the assessment of loneliness, we used a shortened version (6-item version [20]) of the widely used 11-item De Jong Gierveld Loneliness Scale, where participants indicated their agreement on a scale from 1 (strongly agree) to 4 (strongly disagree). The psychometric properties of this scale have been demonstrated in previous research [21]. Higher scores on this scale represent higher levels of loneliness. In our study, Cronbach’s alpha was 0.81 (McDonald’s omega: 0.81).

To measure depressive symptoms, we employed the 15-item version of the Center for Epidemiologic Studies Depression Scale (CES-D) [22]. Two items were recoded, and each item was rated on a scale ranging from 1 (rarely/none of the time) to 4 (most/all of the time), with the value range transformed from 1 to 4 to 0 to 3. The total score on this scale ranged from 0 to 45, indicating the severity of depressive symptoms. The scale’s psychometric properties have been established as favorable in previous studies [23]. In our study, Cronbach’s alpha was 0.84 (McDonald’s omega: 0.86).

Life satisfaction, representing the cognitive evaluation of life, was assessed using the Satisfaction with Life Scale (SWLS) [24], which consisted of five items rated on a five-point scale. The final score was derived by calculating the mean of all items, with higher scores indicating greater life satisfaction. In our study, Cronbach’s alpha was 0.84 (McDonald’s omega: 0.85).

To measure positive (e.g., joy) and negative emotions (e.g., anxiety or anger), we employed the Positive Affect and Negative Affect Schedule (PANAS) [25], consisting of ten items for each subscale. The final score for each subscale ranged from 1 to 5, with higher scores indicating greater positive or negative affect, respectively. In our study, Cronbach’s alpha was 0.87 (McDonald’s omega: 0.87) for the positive affect subscale and 0.85 (McDonald’s omega: 0.85) for the negative affect subscale.

The level of satisfaction with aging was measured using a well-established tool entitled the “Attitudes Toward Own Ageing” (ATOA) subscale, which is part of the Philadelphia Geriatric Morale Scale (PGCMS) [26, 27]. The ATOA subscale consists of five items, with each item rated on a scale of 1 to 4. Three of the five items were recoded. To obtain an overall score, the ratings for all five items were averaged, resulting in a score ranging from 1 to 4. A higher score indicates a greater level of satisfaction with aging. Previous research has confirmed the favorable psychometric properties of this tool [28, 29]. In this study, Cronbach’s alpha was 0.78 (McDonald’s omega also yielded a value of 0.78).

### Independent variables of interest: perceived ageism

Participants were inquired about whether they had experienced discrimination or faced disadvantages as a result of their age within the previous 12 months (responding with a yes or no). The exact wording was: “In the past twelve months, have you been discriminated against due to your age or placed at a disadvantage in relation to others?”. This tool is widely used to evaluate perceived ageism (e.g., [5]).

### Covariates

Consistent with previous studies (e.g., [30]), the regression analysis included sociodemographic and health-related factors as covariates. The sociodemographic variables consisted of gender (men or women), age (measured in years), family situation (single; widowed; divorced; married but living apart from spouse; married and living together with spouse), and education level (classified according to International Standard Classification of Education-97 (ISCED-97) [31] as low [0–2], medium [3–4], or high [5–6] education). Additionally, the regression analysis considered health-related variables, namely self-rated health (rated on a scale from 1 = very good to 5 = very bad, using a single-item tool), and chronic conditions (represented by a count score ranging from 0 to 11, encompassing various conditions: (1) cardiac and circulatory disorders, (2) bad circulation, (3) joint, bone, spinal or back problems, (4) respiratory problems, asthma, shortness of breath, (5) stomach and intestinal problems, (6) cancer, (7) diabetes, (8) gall bladder, liver or kidney problems, (9) bladder problems, (10) eye problems, vision impairment, and (11) ear problems, hearing problems.

### Statistical analysis

The characteristics of the sample were analyzed and presented based on perceived ageism. Additionally, Cohen’s d (an effect size measure of the standardized difference between two means) was calculated to assess the relationship between perceived ageism and psychosocial outcomes. Following Cohen [32], effect sizes of 0.2, 0.5, 0.8 can be classified as small, medium or large, respectively.

Subsequently, multiple linear regressions were conducted to investigate the association between perceived ageism and psychosocial factors (total sample and stratified by age: individuals aged 40 to 64 years; individuals aged 65 years and over). To handle missing data, a full-information maximum likelihood (FIML) approach [33] was employed in the sensitivity analysis. To calculate McDonald’s omega (a measure of international consistency), a quite new Stata tool [34] was used. The internal consistency of the tools used (in terms of Cronbach’s alpha and McDonald’s omega) was reported in the methods section. Statistical significance was defined as *p* < 0.05 in this study. All statistical analyses were performed using Stata 16.1 (Stata Corp., College Station, Texas).

## Results

### Sample characteristics and bivariate analysis

Table 1 provides an overview of the sample characteristics for the analytical sample, which were also stratified based on perceived ageism (presence or absence). In the total sample, the mean age was 68.7 years (standard deviation, SD: 10.1), with the age range spanning from 46 to 98 years. Approximately 50.3% of the individuals were female. In total, 5.6% of the individuals reported ageism (among individuals aged 40 to 64 years: 6.5%; among individuals aged 65 years and over: 5.1%).

**Table 1:** Sample characteristics of the analytical sample (stratified by perceived ageism; German Ageing Survey, wave 7, Germany)

	Absence of perceived ageism	Presence of perceived ageism	Total	*P*-value
Total: N (%)	3933 (94.4)	234 (5.6)	4167 (100.0)	
Sex: N (%)				0.763
- Men	1956 (49.7)	114 (48.7)	2070 (49.7)	
- Women	1977 (50.3)	120 (51.3)	2097 (50.3)	
Age (in years): Mean (SD)	68.7 (10.1)	68.4 (10.2)	68.7 (10.1)	0.597
Education (ISCED-97): N (%)				0.539
- Low education	153 (3.9)	8 (3.4)	161 (3.9)	
- Medium education	1808 (46.0)	100 (42.7)	1908 (45.8)	
- High education	1972 (50.1)	126 (53.8)	2098 (50.3)	
Marital status: N (%)				0.090
- Married, living together with spouse	2774 (70.5)	154 (65.8)	2928 (70.3)	
- Married, living separated from spouse	41 (1.0)	5 (2.1)	46 (1.1)	
- Divorced	356 (9.1)	30 (12.8)	386 (9.3)	
- Widowed	525 (13.3)	27 (11.5)	552 (13.2)	
- Single	237 (6.0)	18 (7.7)	255 (6.1)	
Self-rated health (from 1 = very good to 5 = very bad): Mean (SD)	2.4 (0.8)	2.8 (0.9)	2.4 (0.8)	< 0.001
Count score for chronic illnesses: Mean (SD)	2.7 (2.0)	3.8 (2.4)	2.8 (2.0)	< 0.001
Loneliness: Mean (SD)	1.8 (0.5)	2.1 (0.6)	1.8 (0.5)	< 0.001
Perceived social isolation: Mean (SD)	1.6 (0.5)	2.0 (0.8)	1.6 (0.6)	< 0.001
Depressive symptoms: Mean (SD)	5.9 (5.5)	10.1 (8.2)	6.1 (5.8)	< 0.001
Life satisfaction: Mean (SD)	3.9 (0.7)	3.5 (0.8)	3.9 (0.7)	< 0.001
Positive affect: Mean (SD)	3.6 (0.5)	3.4 (0.6)	3.6 (0.5)	< 0.001
Negative affect: Mean (SD)	2.0 (0.5)	2.3 (0.6)	2.1 (0.5)	< 0.001
Aging satisfaction: Mean (SD)	3.0 (0.5)	2.7 (0.6)	3.0 (0.5)	< 0.001

*Notes* Chi^2^-tests or Oneway ANOVAs were performed, as appropriate (p-values)

Significant differences between individuals reporting the absence of ageism and individuals reporting the presence of ageism were observed across all psychosocial factors as well as across self-rated health and the number of chronic conditions. However, perceived ageism was not associated with gender, age, education and marital status.

For instance, among individuals without perceived ageism, the average level of loneliness was 1.8 (SD: 0.5), whereas the average level of loneliness was 2.1 (SD: 0.6) among individuals with perceived ageism. Moreover, in the first group, the average depressive symptoms score equaled 5.9 (SD: 5.5) and it equaled 10.1 (SD: 8.2) in the second group. Further details are displayed in Table 1.

Notably, the effect size (Cohen’s d, in absolute values) for the differences in the psychosocial factors between individuals with and without perceived ageism were as follows: d = 0.71 (for loneliness), d = 0.73 (for perceived social isolation), d = 0.73 (for depressive symptoms), d = 0.66 (for life satisfaction), d = 0.31 (for positive affect), d = 0.61 (for negative affect), and d = 0.61 (for aging satisfaction).

### Regression analysis

The results of the multiple linear regressions (with listwise deletion), presented in Table 2, examined the relationship between perceived ageism (presence or absence) and the psychosocial outcomes. The models were adjusted for various covariates including age, sex, family status, education, employment status, self-rated health, number of chronic conditions. It is worth noting that the proportion of missing data very slightly varied depending on the outcome used. Thus, the number of individuals included in the regression model varied from 4,153 to 4,178 (see Table 2).

**Table 2:** Perceived ageism and psychosocial factors among the total sample. Results of multiple linear regressions (German Ageing Survey, wave 7, Germany)

	Loneliness	Perceived social isolation	Depressive symptoms	Life satisfaction	Positive affect	Negative affect	Aging satisfaction
Perceived ageism: Yes (Ref.: No)	0.29***	0.32***	2.68***	-0.28***	-0.06	0.21***	-0.19***
	(0.04)	(0.05)	(0.47)	(0.05)	(0.04)	(0.04)	(0.04)
Covariates^†^	✓	✓	✓	✓	✓	✓	✓
Individuals	4,167	4,164	4,178	4,153	4,155	4,155	4,154
R^2^	0.09	0.11	0.24	0.18	0.14	0.11	0.27

Comments: Unstandardized beta coefficients are shown. Robust standard errors are shown in parentheses. *** *p* < 0.001, ** *p* < 0.01, * *p* < 0.05, + *p* < 0.10

^†^ Covariates include sex, gender, family status, education, employment status, chronic illnesses and self-rated health

Regressions showed that the presence of perceived ageism was significantly associated with unfavorable psychosocial outcomes (i.e., higher loneliness: β = 0.29, *p* < 0.001; higher perceived social isolation: β = 0.32, *p* < 0.001; more depressive symptoms: β = 2.68, *p* < 0.001; lower life satisfaction: β=-0.28, *p* < 0.001; higher negative affect: β = 0.21, *p* < 0.001; lower aging satisfaction: β=-0.19, *p* < 0.001), except for positive affect (β=-0.06, *p* = 0.10). Stratified by age group (i.e., individuals 40 to 64 years; individuals aged 65 years and over, see Tables 3 and 4), comparable results were obtained in terms of significance. Please see the Tables 3 and 4 for further details.

**Table 3:** Perceived ageism and psychosocial factors among individuals aged 40 to 64 years. Results of multiple linear regressions (German Ageing Survey, wave 7, Germany)

	Loneliness	Perceived social isolation	Depressive symptoms	Life satisfaction	Positive affect	Negative affect	Aging satisfaction
Perceived ageism: Yes (Ref.: No)	0.20***	0.22**	1.99**	-0.29***	-0.06	0.20**	-0.23***
	(0.06)	(0.07)	(0.75)	(0.07)	(0.06)	(0.07)	(0.06)
Covariates^†^	✓	✓	✓	✓	✓	✓	✓
Individuals	1,474	1,472	1,475	1,469	1,470	1,470	1,472
R²	0.12	0.14	0.25	0.26	0.18	0.12	0.28

Comments Unstandardized beta coefficients are shown. Robust standard errors are shown in parentheses. *** *p* < 0.001, ** *p* < 0.01, * *p* < 0.05, + *p* < 0.10

^†^ Covariates include sex, gender, family status, education, employment status, chronic illnesses and self-rated health

**Table 4:** Perceived ageism and psychosocial factors among individuals aged 65 years and over. Results of multiple linear regressions (German Ageing Survey, wave 7, Germany)

	Loneliness	Perceived social isolation	Depressive symptoms	Life satisfaction	Positive affect	Negative affect	Aging satisfaction
Perceived ageism: Yes (Ref.: No)	0.36***	0.38***	3.20***	-0.27***	-0.07	0.22***	-0.16***
	(0.05)	(0.06)	(0.60)	(0.06)	(0.05)	(0.05)	(0.05)
Covariates^†^	✓	✓	✓	✓	✓	✓	✓
Individuals	2,693	2,692	2,703	2,684	2,685	2,685	2,682
R²	0.09	0.09	0.25	0.15	0.12	0.10	0.25

Comments Unstandardized beta coefficients are shown. Robust standard errors are shown in parentheses. *** *p* < 0.001, ** *p* < 0.01, * *p* < 0.05, + *p* < 0.10

^†^ Covariates include sex, gender, family status, education, employment status, chronic illnesses and self-rated health

It is noteworthy that the results obtained from regressions with both listwise deletion and FIML were nearly identical. Therefore, the results from the FIML approach were not presented here, but are displayed in the Supplementary Tables 1 to 3.

## Discussion

Based on nationally representative data, our objective was to investigate the association between ageism and psychosocial outcomes among individuals in middle and older adulthood during the COVID-19 pandemic. Our key findings: The effect sizes (Cohen’s d) for the link between perceived ageism and psychosocial factors were mainly moderate to large. Regressions showed that the presence of perceived ageism was significantly associated with unfavorable psychometric outcomes (except for positive affect). Stratified by age group, comparable results were obtained. Our current research expands upon our existing knowledge, which is partly based on small, selective samples and mainly based on pre-pandemic studies, by including a nationally representative sample during the pandemic.

With regard to subjective well-being (covering positive and negative affect as well as life satisfaction), we found that perceived ageism was significantly associated with life satisfaction and negative affect, but not positive affect. A recent study also found an association between perceived ageism and lower life satisfaction among community-dwelling individuals aged 60 years and over from the Grand Duchy of Luxembourg in June 2020 (i.e., during the Covid-19 pandemic) [16]. A former study found that a positive outlook on aging significantly mediated the link with life satisfaction and may thus help explaining such an association [35]. It appears a bit puzzling that perceived ageism was not significantly associated with positive affect. A potential explanation may be that individuals may only adapt (in terms of positive emotions) to perceived ageism (more general, please see: [36]). Certain positive emotions such as pride may be rather stable within individuals over time.

The link between the presence of perceived ageism and more depressive symptoms which was found in our study aligns previous research summarized in a recent systematic review [2]. Prior studies have found that, among other things, a low purpose in life (mediator) could explain the association between perceived ageism and depressive symptoms based on data from the Health and Retirement Study [12]. This is in line with Beck’s cognitive theory of depression [37]. Following this theory, encountering ageism could serve as a catalyst for a diminished sense of purpose in life. This in turn could lead to increases in depressive symptoms [12].

Our current study showed an association between perceived ageism and both higher loneliness and perceived social isolation levels. Comparable studies are extremely scarce. However, a former study [11] showed an association between negative age stereotypes (a measure at least somewhat correlated with perceived ageism) and higher loneliness levels. We assume that perceived ageism could act as a stressor (see: [38]) which could lead, for example, to social withdrawal. This could explain the link between perceived ageism and loneliness as well as social isolation.

In this study, we found an association between perceived ageism and aging satisfaction. Another study from Luxembourg found an association between perceived ageism (in June 2020) and subsequent self-perceptions of aging (as physical decline and social loss; in October 2020) [39]. We assume that the distress associated with perceived ageism may contribute to a reduced satisfaction with aging [40] – which could explain our current findings.

We would like to emphasize both the strengths and caveats of our current research. Firstly, we utilized a substantial and nationally representative sample collected during the Covid-19 pandemic, ensuring the robustness of our data. Additionally, we employed well-established and reliable tools to quantify our outcome measures, enhancing the validity of our findings. To address missing data, we additionally applied a FIML approach. However, it is important to acknowledge certain shortcomings of our study. The use of a cross-sectional design limits our ability to determine causality and establish the direction of relationships. For example, it is plausible that more depressive symptoms contribute to feelings of ageism. Another example: Feelings of social isolation may lead to perceived ageism. However, we assume that this is rarely the case. It is important to acknowledge that the evaluation of ageism relied on a single-question assessment, which may not capture the entire spectrum of experiences related to discrimination based on age. Similarly, it is essential to recognize that perceiving discrimination does not necessarily indicate that actual discrimination has occurred. However, in fact, individuals were asked about their actual experiences of ageism in the last 12 months. Additionally, a slight sample selection bias was detected in the DEAS study, which may impact the generalizability of our results [18]. Furthermore, our study focused exclusively on individuals residing in non-institutionalized settings, which restricts the extent to which our findings can be applied to populations living in institutionalized settings. During the pandemic media attention largely focused on such individuals. These individuals may therefore face an additional burden (also due to the increased risk of a severe course of the disease and experiences of ageism [16].

## Conclusion

In our study, perceived ageism was associated with unfavorable psychosocial outcomes. This knowledge can help reduce vulnerability to negative psychosocial factors in people in the middle and later years of life.

### Electronic supplementary material

Below is the link to the electronic supplementary material.

Supplementary Material 1

## Data Availability

The data used in this study are third-party data. The anonymized data sets of the DEAS (1996, 2002, 2008, 2011, 2014, 2017, 2020, 2020/2021) are available for secondary analysis. The data has been made available to scientists at universities and research institutes exclusively for scientific purposes. The use of data is subject to written data protection agreements. Microdata of the German Ageing Survey (DEAS) is available free of charge to scientific researchers for non-profitable purposes. The FDZ-DZA provides access and support to scholars interested in using DEAS for their research. However, for reasons of data protection, signing a data distribution contract is required before data can be obtained. Please see for further information (data distribution contract): https://www.dza.de/en/research/fdz/access-to-data/formular-deas-en-english. Please see for further details: https://www.dza.de/en/research/fdz/contact-and-support

## Abbreviations

ATOA: Attitudes Toward Own Ageing
BMFSFJ: Federal Ministry for Family Affairs, Senior Citizens, Women, and Youth
CAPI: Computer-Assisted Personal Interviewing
CES-D: Center for Epidemiologic Studies Depression Scale
DEAS: German Ageing Survey
FIML: Full-Information Maximum Likelihood
ISCED: International Standard Classification of Education
PANAS: Positive Affect and Negative Affect Schedule
PGCMS: Philadelphia Geriatric Morale Scale
SD: Standard deviation
SWLS: Satisfaction with Life Scale
VIF: Variance Inflation Factor
