# Supplementary material for: Perceived ageism and psychosocial outcomes during the COVID-19 pandemic
Source: Arch Public Health. 2024 May 10;82:69. doi: 10.1186/s13690-024-01297-2 (PMC11088088; doi:10.1186/s13690-024-01297-2)
Supplement: Supplementary file 1 — Supplementary Material 1 [file 13690_2024_1297_MOESM1_ESM.docx]

**Supplementary Table 1.** Perceived ageism and psychosocial factors among the total sample. Results of multiple linear regressions (with FIML to address missings; German Ageing Survey, wave 7, Germany).

|  | Loneliness | Perceived social isolation | Depressive symptoms | Life satisfaction | Positive affect | Negative affect | Aging satisfaction |
| --- | --- | --- | --- | --- | --- | --- | --- |
|  |  |  |  |  |  |  |  |
|  |  |  |  |  |  |  |  |
| Perceived ageism: Yes (Ref.: No) | 0.29*** | 0.33*** | 2.78*** | -0.29*** | -0.06 | 0.22*** | -0.19*** |
|  | (0.04) | (0.05) | (0.48) | (0.05) | (0.04) | (0.04) | (0.04) |
|  |  |  |  |  |  |  |  |
| Covariates^†^ | 🗸 | 🗸 | 🗸 | 🗸 | 🗸 | 🗸 | 🗸 |
|  |  |  |  |  |  |  |  |
|  |  |  |  |  |  |  |  |
| Individuals | 4,344 | 4,340 | 4,356 | 4,326 | 4,327 | 4,327 | 4,330 |
| R² | 0.10 | 0.12 | 0.25 | 0.19 | 0.14 | 0.12 | 0.29 |

Comments: Unstandardized beta coefficients are shown. Robust standard errors are shown in parentheses. *** p<0.001, ** p<0.01, * p<0.05, + p<0.10.

^†^ Covariates include sex, gender, family status, education, employment status, chronic illnesses and self-rated health.

**Supplementary Table 2.** Perceived ageism and psychosocial factors among individuals aged 40 to 64 years. Results of multiple linear regressions (with FIML to address missings; German Ageing Survey, wave 7, Germany).

|  | Loneliness | Perceived social isolation | Depressive symptoms | Life satisfaction | Positive affect | Negative affect | Aging satisfaction |
| --- | --- | --- | --- | --- | --- | --- | --- |
|  |  |  |  |  |  |  |  |
|  |  |  |  |  |  |  |  |
| Perceived ageism: Yes (Ref.: No) | 0.20*** | 0.24*** | 2.08** | -0.30*** | -0.05 | 0.21** | -0.23*** |
|  | (0.06) | (0.07) | (0.76) | (0.07) | (0.06) | (0.07) | (0.06) |
|  |  |  |  |  |  |  |  |
| Covariates^†^ | 🗸 | 🗸 | 🗸 | 🗸 | 🗸 | 🗸 | 🗸 |
|  |  |  |  |  |  |  |  |
|  |  |  |  |  |  |  |  |
| Individuals | 1,520 | 1,518 | 1,521 | 1,514 | 1,515 | 1,515 | 1,518 |
| R² | 0.13 | 0.16 | 0.26 | 0.26 | 0.19 | 0.12 | 0.29 |

Comments: Unstandardized beta coefficients are shown. Robust standard errors are shown in parentheses. *** p<0.001, ** p<0.01, * p<0.05, + p<0.10.

^†^ Covariates include sex, gender, family status, education, employment status, chronic illnesses and self-rated health.

**Supplementary Table 3.** Perceived ageism and psychosocial factors among individuals aged 65 years and over. Results of multiple linear regressions (with FIML to address missings; German Ageing Survey, wave 7, Germany).

|  | Loneliness | Perceived social isolation | Depressive symptoms | Life satisfaction | Positive affect | Negative affect | Aging satisfaction |
| --- | --- | --- | --- | --- | --- | --- | --- |
|  |  |  |  |  |  |  |  |
|  |  |  |  |  |  |  |  |
| Perceived ageism: Yes (Ref.: No) | 0.36*** | 0.40*** | 3.31*** | -0.27*** | -0.07 | 0.22*** | -0.16*** |
|  | (0.05) | (0.07) | (0.62) | (0.06) | (0.05) | (0.05) | (0.05) |
|  |  |  |  |  |  |  |  |
| Covariates^†^ | 🗸 | 🗸 | 🗸 | 🗸 | 🗸 | 🗸 | 🗸 |
|  |  |  |  |  |  |  |  |
|  |  |  |  |  |  |  |  |
| Individuals | 2,824 | 2,822 | 2,835 | 2,812 | 2,812 | 2,812 | 2,812 |
| R² | 0.09 | 0.11 | 0.27 | 0.15 | 0.12 | 0.11 | 0.26 |

Comments: Unstandardized beta coefficients are shown. Robust standard errors are shown in parentheses. *** p<0.001, ** p<0.01, * p<0.05, + p<0.10.

^†^ Covariates include sex, gender, family status, education, employment status, chronic illnesses and self-rated health.
